# Multivariate genetic analyses test for the presence of a general ‘*n*’ factor underlying neurodevelopmental conditions

**DOI:** 10.1002/jcv2.70168

**Published:** 2026-09-29

**Authors:** Morgan James Morgan, Margherita Malanchini, Elise B. Robinson, Angelica Ronald

**Affiliations:** ^1^ School of Psychology, Faculty of Health & Medical Sciences University of Surrey Guildford UK; ^2^ School of Biological and Behavioural Sciences Queen Mary University of London London UK; ^3^ The Broad Institute of MIT and Harvard Cambridge Massachusetts USA

**Keywords:** ADHD, autism, genetics, GenomicSEM, neurodevelopment

## Abstract

**Background:**

Neurodevelopmental conditions often emerge early in life and have substantial genetic influences. There are significant genetic correlations between neurodevelopmental conditions, such as between autism spectrum disorder (Autism) and attention deficit‐hyperactivity disorder (ADHD), and they are listed together in diagnostic manuals. Here we assess whether there is a single underlying genetic neurodevelopmental (‘*n*’) factor for neurodevelopmental conditions.

**Methods:**

Genetic summary statistics of neurodevelopmental conditions, or their proxy traits, were used to model the genetic relationship between neurodevelopmental conditions. Summary statistics contain the effect sizes of each variant's association with a neurodevelopmental condition from a genome‐wide association study. Single and multiple common factor models were fitted to the data using genomic structural equation modelling.

**Results:**

A single common factor model did not provide a good fit for the genetic architecture of neurodevelopmental conditions. A two‐factor model fitted the data best. It was also found that the common genetic variation associated with a trait representing motor development, namely age at onset of walking, was negatively and positively associated with ADHD and Autism, respectively, suggesting some genetic differentiation between these two common neurodevelopmental conditions.

**Conclusion:**

These results provide insight into the genetic architecture of neurodevelopmental conditions and their inter‐related structure. Data on measured common genetic variation, as discovered from recent genome‐wide association studies, does not support the hypothesis that all neurodevelopmental conditions fall into a single ‘*n*’ factor.

## Key Points

**What's Known?**

- Neurodevelopmental conditions such as attention deficit‐hyperactivity disorder (ADHD), autism and Tourette's show high rates of co‐occurrence underpinned by shared genetic architecture.

**What's New?**

- We have shown that a single ‘*n*’ factor does not adequately capture genetic overlap across neurodevelopmental conditions.
- We reveal that the early motor milestone age at onset of walking shows opposite genetic associations with autism and ADHD.

**What's Relevant?**

- Although grouped together as neurodevelopmental conditions, our results using models of genetic data do not support the model of a single genetic factor underlying neurodevelopmental conditions
- These findings support dimensional frameworks for classification and care, given the considerable shared generic liabilities across neurodevelopmental conditions

## INTRODUCTION

Neurodevelopmental conditions, referred to as neurodevelopmental disorders in DSM‐5, are a group of conditions that manifest during the developmental period and often impact personal, social, academic, or occupational functioning (APA, 2022). These conditions affect approximately 15% of children and adolescents globally (Francés et al., 2022) and encompass a range of impairments in cognition, communication, and adaptive behaviour. These conditions, which include intellectual development disorders, communication disorders, Autism, attention deficit‐hyperactivity disorder (ADHD), specific learning disorders and motor disorders, pose significant challenges for diagnosis, treatment, and research due to heterogeneity at both the genetic and phenotypic levels (Zamora‐Moratalla et al., 2021). The high rates of co‐occurrence and overlapping symptoms among neurodevelopmental conditions complicate clinical assessments and suggest that current diagnostic categories may not fully capture the underlying nature of these conditions (Astle et al., 2022; Michelini et al., 2024; Morris‐Rosendahl & Crocq, 2020; Reiersen & Todd, 2008).

A growing body of phenotypic research indicates that neurodevelopmental conditions are not best understood as entirely separate categories but rather as overlapping and heterogeneous presentations that often cluster within broader transdiagnostic dimensions (Astle et al., 2022; Michelini et al., 2024). Recent work has formalised this idea as a ‘neurodevelopmental spectrum’, defined as a broad latent dimension capturing the overlapping features, early developmental trajectories, and shared liabilities of neurodevelopmental conditions (Michelini et al., 2025). In a recent study, hierarchical factor modelling in a large longitudinal twin sample demonstrated that a broad phenotypic neurodevelopmental spectrum can be identified across childhood and adolescence (Michelini et al., 2025). In some literature, schizophrenia is considered a neurodevelopmental condition, however, because the present study was pre‐registered to focus on the DSM‐5‐TR neurodevelopmental disorders, we did not include schizophrenia.

Most individuals with a neurodevelopmental condition diagnosis have a second neurodevelopmental or psychiatric condition. A systematic review of studies investigating the prevalence of neurodevelopmental conditions found that these studies reported co‐occurrence of more than one condition as the norm (Francés et al., 2022). For example, a 2018 study showed that of children with one neurodevelopmental condition, 22% have a second neurodevelopmental condition, and 58% have a psychiatric condition (not including neurodevelopmental conditions) (Hansen et al., 2018). This rate of co‐occurrence is particularly high for autism in combination with ADHD or developmental co‐ordination disorder (DCD) with the co‐occurrence estimated in a meta‐analysis to be 37% and 87% respectively (Micai et al., 2023).

There is evidence from twin and molecular genetic studies that there are significant genetic influences on neurodevelopmental conditions (see Table 1).

**TABLE 1:** Heritability of neurodevelopmental conditions.

Conditions	Twin heritability, %	SNP‐based heritability
Autism	64–91	14%
ADHD	74	20%
Intellectual disability	86	N/A
Communication disorders	64	32%
Specific learning disorders	62	30%
Motor disorders	74	N/A
NDCs	66	19%

*Note*: Broad sense heritability as estimated from meta‐analyses of twin studies (for autism—Tick et al., 2016, for ADHD—Faraone & Larsson, 2019, for all other conditions—Gidziela et al., 2023). SNP‐based heritability from meta‐analyses of the GWAS (Gidziela et al., 2023). Estimates for intellectual disability and motor disorders were unavailable due to the lack of sufficient studies for those conditions.

Abbreviations: ADHD, attention deficit‐hyperactivity disorder; GWAS, genome‐wide association studies; NDCs, neurodevelopmental conditions; SNP, single‐nucleotide polymorphism.

A genome‐wide association study (GWAS) is a design which investigates the association between common genetic variants and specific traits, studying the whole genome in a single study (Willoughby et al., 2023). GWAS studies of neurodevelopmental conditions have identified the heritability associated with this common genetic variance, termed single‐nucleotide polymorphism (SNP) heritability. Table 1 above sets out the SNP‐based heritabilities for neurodevelopmental conditions. Although studies of intellectual disability and motor disorders were unavailable, there are well‐powered GWAS of childhood cognitive performance, which could be used as a proxy trait for intellectual disability, and of age at onset of walking, a major gross motor milestone that is used as an indicator of early gross motor development relevant to developmental motor variation and could be used as a proxy trait for DCD, a neurodevelopmental motor disorder (Benyamin et al., 2014; Gui et al., 2025). A recent meta‐analysis showed that DCD is significantly associated with delayed walking and this association is largest compared to other neurodevelopmental conditions (Bowler et al., 2024). Childhood cognitive performance and age at onset of walking show significant SNP heritabilities of 22%–46% and 24%, respectively. Available polygenic scores for neurodevelopmental conditions also explain significant, though modest, variance in related neurodevelopmental traits and outcomes. (for reviews of autism and ADHD polygenic scores, see de Wit et al., 2025; Ronald et al., 2021).

Furthermore, twin studies and molecular genetic studies both suggest there are overlapping genetic influences across neurodevelopmental conditions (Gidziela et al., 2023; Ronald & Hoekstra, 2011). This suggests that these conditions may share common genetic factors. A recent meta‐analysis reported genetic correlations between all neurodevelopmental conditions to be 0.39 from GWAS data and 0.47 from twin or family studies (Gidziela et al., 2023). Early twin and family studies have shown that co‐occurring phenotypes such as autistic and ADHD traits, correlate genetically to a moderate degree (*r*_g_ = .56; *r*_g_ is a statistic that estimates the extent to which the same genetic variants influence two different traits) (Ronald et al., 2008), with a later Swedish twin analysis confirming genetic correlations between the traits of these conditions at up to *r*_g_ = .64 (Polderman et al., 2014). Using measured DNA, SNP‐based methods detect significant cross‐disorder genetic correlations: the Autism GWAS from the Psychiatric Genomics Consortium (PGC) reported common‐variant overlap spanning Autism and ADHD of *r*_g_ = .36 (Grove et al., 2019), a pattern reinforced by a PGC GWAS of ADHD which showed an *r*_g_ of .42 between Autism and ADHD (Demontis et al., 2023). Regarding Tourette's syndrome and dyslexia, recent studies have shown substantial genetic correlations with autism and ADHD, with *r*_g_ = .13 for autism and Tourette's syndrome, *r*_g_ = .23 for ADHD and Tourette's syndrome, *r*_g_ = .40 for ADHD and dyslexia, and *r*_g_ = .41 for autism and dyslexia (Ciulkinyte et al., 2025; Grotzinger et al., 2026). Both these studies also showed significant genetic correlations with childhood intellectual ability (*r*_g_ = .23 for Autism and *r*_g_ = −.41 for ADHD). Although the multivariate genetic literature has focused most on Autism and ADHD, cross‐disorder research also indicates broader overlaps across neurodevelopmental and related psychiatric phenotypes (Gidziela et al., 2023; Grotzinger et al., 2026). Collectively, these results suggest that observed clinical co‐occurrence is at least partly due to overlapping genetic architecture.

In the mental health literature, it has been proposed that psychiatric conditions, often categorised as distinct entities, are better understood as existing on a continuum across a shared general dimension of psychopathology, captured by a ‘*p*’ factor (Caspi et al., 2014; Ronald, 2019). Studies in both adults (Caspi et al., 2014; Lahey et al., 2012) and children (Martel et al., 2017) have used confirmatory factor analysis (CFA) to identify that a single ‘*p*’ factor model best fits psychiatric conditions and psychopathological symptoms in general. The ‘*p*’ factor has also been investigated using genetic data. Principal components analysis in children and adolescents has provided strong evidence for a heritable genetic basis of the proposed ‘*p*’ factor with a heritability estimate of 50%–60% (Allegrini et al., 2020; Selzam et al., 2018). Although other studies do not find evidence to support a genetic ‘*p*’ factor underlying psychiatric conditions (Grotzinger et al., 2022).

Given the observed high rates of co‐occurrence and the high heritabilities of neurodevelopmental conditions, the present study aimed to determine whether a single, overarching genetic factor, analogous to the ‘*p*' factor in psychopathology, underlay neurodevelopmental conditions, which we termed the neurodevelopmental ‘*n*’ factor. Additionally, considering the developmental importance of early motor milestones, we examined whether common genetic variation associated with age at onset of walking showed significant genetic associations with each neurodevelopmental condition examined when analysed independently of the other clinical phenotypes. This analysis aimed to investigate whether the associations reported in previous meta‐analyses showing that gross motor development is phenotypically associated with neurodevelopmental conditions (Bowler et al., 2024) are also present at the genetic level when assessed independently. Similarly, given the shared cognitive–linguistic basis of learning difficulties, we evaluated whether age at onset of walking, language acquisition, and intelligence are best captured by a single latent genetic factor.

Our aim was to investigate the underlying common variant genetic architecture of neurodevelopmental conditions. Our pre‐registered hypotheses were that: (1) there are positive and significant common variant genetic associations between neurodevelopmental conditions, (2) a single‐factor latent genetic model best fits the common genetic architecture of neurodevelopmental conditions when modelled together, (3) when the age at the onset of walking is analysed separately from neurodevelopmental conditions, it shows a significant conditional genetic association with each of the neurodevelopmental conditions, and (4) phenotypes relating to learning disabilities, specifically the age at the onset of walking, language acquisition, and intelligence, fit a single latent genetic factor model. Please note that hypothesis 3, as set out above, has been amended in response to a reviewer request.

## METHODS

Our analyses were pre‐registered on the Open Science Framework (https://osf.io/uges3). Any deviations from the pre‐registration are noted explicitly in the text.

### Phenotype selection

Phenotypes included in this study were those identified as neurodevelopmental conditions according to the DSM‐5‐TR (APA, 2022). The DSM‐5‐TR category was employed here as an operational definition for phenotype selection because it offers a clinically meaningful set of conditions, though it is not considered a biologically definitive or ideal classification. Accordingly, the aim of the present analyses was to test whether their common variant genetic architecture is better captured by a single shared dimension or by a more differentiated structure. Where direct GWAS data were unavailable or insufficiently powered, related developmental indicators were chosen to represent the relevant domain (e.g., childhood intelligence as a proxy phenotype for intellectual disability). This approach aimed to maintain representation of key neurodevelopmental domains while recognising that these indicators are not equivalent to clinical diagnoses. Age at onset of walking was included as an indicator of early gross motor development relevant to developmental motor variation and that is associated with DCD (Bowler et al., 2024), rather than as a direct diagnostic equivalent of DCD. This decision was made because walking onset is a major gross motor milestone that can be reliably measured on a large scale and has been shown to reflect heritable variation in early motor development and consistent associations with DCD (Bowler et al., 2024; Gui et al., 2025). Similarly, childhood intelligence and early expressive vocabulary were regarded as developmental indicator phenotypes for domains related to intellectual disability and language, especially where adequately powered GWAS of the relevant clinical diagnoses were not available. Phenotypes and their corresponding GWAS studies are detailed in Table 2.

**TABLE 2:** GWAS studies of neurodevelopmental conditions or their proxies.

Phenotype	Study	Discovery sample size
Autism spectrum disorder	PGC Autism (unpublished)	Cases 38,717; controls 232,735
Attention deficit/hyperactivity disorder	Demontis et al. (2023)	Cases 38,691; controls 186,843
Tourette's syndrome	Yu et al. (2019)	Cases 4819; controls 9488
Developmental co‐ordination disorder (proxy age at onset of walking)	Gui et al. (2025)	70,560
Language disorder (proxy early expressive vocabulary)	Verhoef et al. (2024)	19,296
Intellectual disability (proxy childhood intelligence)	Benyamin et al. (2014)	17,989

*Note*: Discovery sample size refers to number of individuals genotyped for GWAS either in total or split between cases and controls. For the childhood intelligence proxy phenotype, higher values indicate higher intellectual ability and therefore lower liability for intellectual disability. The data from PGC Autism are unpublished data from a 2024 freeze of an upcoming GWAS.

Abbreviations: GWAS, genome‐wide association studies; PGC, Psychiatric Genomics Consortium.

### Summary statistics for neurodevelopmental conditions

GWAS summary statistics were selected based on the largest available sample sizes for each neurodevelopmental condition. Data were retrieved from publicly accessible GWAS repositories indexing the specific phenotype of interest, including GWASAtlas (https://atlas.ctglab.nl/), GWAS Catalogue (http://www.ebi.ac.uk/gwas/downloads/summary‐statistics), GWAS Central (https://www.gwascentral.org), and the Psychiatric Genomics Consortium (PGC; https://www.med.unc.edu/pgc/download‐results/). Selected studies and their details are summarised in Table 2 (and the SNP‐based heritabilities for each condition or their proxy trait are set out in Supporting Information S1: Table S2).

### GenomicSEM

GenomicSEM is a statistical framework that extends structural equation modelling to the analysis of GWAS summary statistics, enabling researchers to investigate relationships between multiple traits at the genetic level (Grotzinger et al., 2019). This approach allows for the estimation of latent genetic factors that underlie the observed correlations between neurodevelopmental conditions, providing insights into shared genetic pathways and etiological mechanisms. The estimation of covariances was carried out using LD score regression (LDSC) (Bulik‐Sullivan et al., 2015) and GenomicSEM, then used to evaluate the fit of the models to that covariance structure. Genomic‐SEM is robust to sample overlap and differences in sample sizes, minimising biases associated with these factors (Grotzinger et al., 2019).

### Data preparation and quality control

Prior to analysis, conversion and quality control steps were performed to prepare the summary statistics for LDSC. Summary statistics were converted to produce z‐scores and filtered using the munge function in GenomicSEM. Data were aligned to HapMap 3 SNPs and SNPs not in the reference file were discarded. In accordance with standard thresholds, SNPs with imputation INFO scores of 0.9 or below and those with a minor allele frequency of 0.01 or below were removed. The sample sizes for each condition, measured on a binary basis (being autism, ADHD, and Tourette's syndrome), were calculated as the sum of the effective sample sizes for each cohort in the discovery sample of the GWAS (Supporting Information S1: Table S1), where it is a meta‐analysis across multiple cohorts (Grotzinger et al., 2023). Because GWAS discovery sample sizes and SNP‐based heritabilities varied substantially across phenotypes, weaker genetic correlations and downstream factor loadings were interpreted cautiously.

### LDSC and genetic correlations

Prior to model fitting, estimation of pairwise genetic correlations between the summary statistics for the neurodevelopmental conditions (or proxies) was carried out. LDSC, a method that regresses each SNP on its linkage disequilibrium score to estimate SNP‐based heritability and a genetic correlation with other traits, was employed (Bulik‐Sullivan et al., 2015).

To test hypothesis 1, a multivariate version of LDSC within GenomicSEM was used to estimate the genetic correlations between neurodevelopmental conditions and to create the covariance matrix required for GenomicSEM analyses. The LDSC function requires the munged summary statistics as well as sample and population prevalences from the GWAS case‐control studies. These prevalences were taken from the relevant GWASs and from recent studies of the prevalences of neurodevelopmental conditions (Bougeard et al., 2021; Popit et al., 2024; Xiong et al., 2024). LDSC was run with LD scores from a European ancestry reference panel from HapMap3. The estimated genetic covariance matrix and the accompanying sampling covariance matrix served as the foundation for subsequent GenomicSEM analyses.

### Exploratory factor analyses

To test hypothesis 2, exploratory factor analyses (EFA) were conducted using the LDSC generated genetic covariance matrix to assess different factor models. This was carried out using the factanal function in the stats package in R. To avoid overfitting, the dataset was split into even and odd chromosome data, and LDSC was carried out on both sets of data. Given that all of the conditions are highly polygenic, the same models should fit both sets of data (Grotzinger et al., 2022). As such, models were based on the results of the EFA of odd chromosome data and then tested using CFA in the even chromosome data (see supplementary notes in Grotzinger et al., 2022).

### Confirmatory factor analysis

Models from the EFA were chosen using two criteria: first, that all models included the same set of indicators (i.e., all of the neurodevelopmental conditions) to allow direct comparison across models; and second, that each factor had at least three indicators to ensure the model was locally identified. Models were chosen using the criteria that all indicators with a loading in the EFA above 0.2 (against one or more latent factor) were retained and where any indicator did not have a loading above 0.2 against any latent factor then only the single highest loading was kept. To enable local identification of the models, if any latent factor did not have at least three indicators with loadings based on the aforementioned criteria, the next highest loading indicators were added to make up three. CFA in GenomicSEM using the usermodel function was employed to evaluate the fit of the proposed factor models to the observed genetic covariance matrix. We evaluated the model fit indices, namely, the akaike information criterion (AIC, where lower values indicate a better fit, see Akaike, 1987), comparative fit index (CFI, with values above 0.95 suggesting an excellent fit, see Bentler, 1990) and standardised root mean square residual (SRMR), where values below 0.08 indicate a good fit, see Bentler (1995), to assess the overall adequacy of competing models (Hu & Bentler, 1999).

Based on the results of the EFA, we assessed both common factor models with 1 latent factor and 2 latent factors, as well as a bi‐factor model with a general latent factor in addition to two common latent factors. All these models allowed for correlated factors to permit for the estimation of genetic correlations between latent factors of neurodevelopmental conditions. The Cholesky model was included as an additional, non‐preregistered analysis. This was motivated by the observation that childhood intelligence showed different patterns of association with the two strongest neurodevelopmental indicators in our data, being positively associated with Autism and negatively associated with ADHD. We therefore used a Cholesky decomposition to assess whether shared genetic variance remained among the neurodevelopmental phenotypes after accounting for variance associated with childhood intelligence. The Cholesky model was used as a restricted variance‐decomposition approach. A Cholesky model is a method of partitioning variation across multiple traits, where the first latent factor explains commonality shared by all traits, the second latent factor explains commonality shared by all but the first trait, and so on. These models did not permit for correlations between the intellectual disability factor and the remaining factor or factors. To test hypothesis 4, we assessed the fit of a single common latent factor model.

### Regression models

To test hypothesis 3, regression models using the usermodel function within GenomicSEM were used to estimate the independent effects of the genetic variance associated with each of the neurodevelopmental conditions on the other neurodevelopmental conditions. This analysis aimed to determine whether a particular condition was a significant genetic predictor of another neurodevelopmental condition independently of the influence of other conditions.

## RESULTS

### Genetic correlations between conditions

In line with previous studies, LDSC revealed extensive but selective genetic overlap among the six phenotypes (Figure 1). ADHD exhibited the largest number of significant correlations, with positive significant genetic correlations with Autism (*r*_g_ = .43, *p* = 4.1 × 10^−14^) and Tourette (*r*_g_ = .18, *p* = 2.0 × 10^−3^). ADHD's genetic correlation with the DCD proxy (age at onset of walking) and the intellectual disability proxy (childhood intelligence) were both significant and negative (*r*_g_ = −.17, *p* = 6.3 × 10^−5^), (*r*_g_ = −.33, *p* = 8.8 × 10^−5^), respectively. The genetic correlation was significant between Autism and the intellectual disability proxy (childhood intelligence) (*r*_g_ = .32, *p* = 4.7 × 10^−4^). Finally, the genetic correlation between the DCD proxy (age at onset of walking) and the intellectual disability proxy (childhood intelligence) was significant and positive (*r*_g_ = .23, *p* = 1.3 × 10^−2^). Other pairwise correlations did not survive false discovery rate (FDR) adjusted multiple‐testing corrections (Supporting Information S1: Table S3).

**FIGURE 1:**
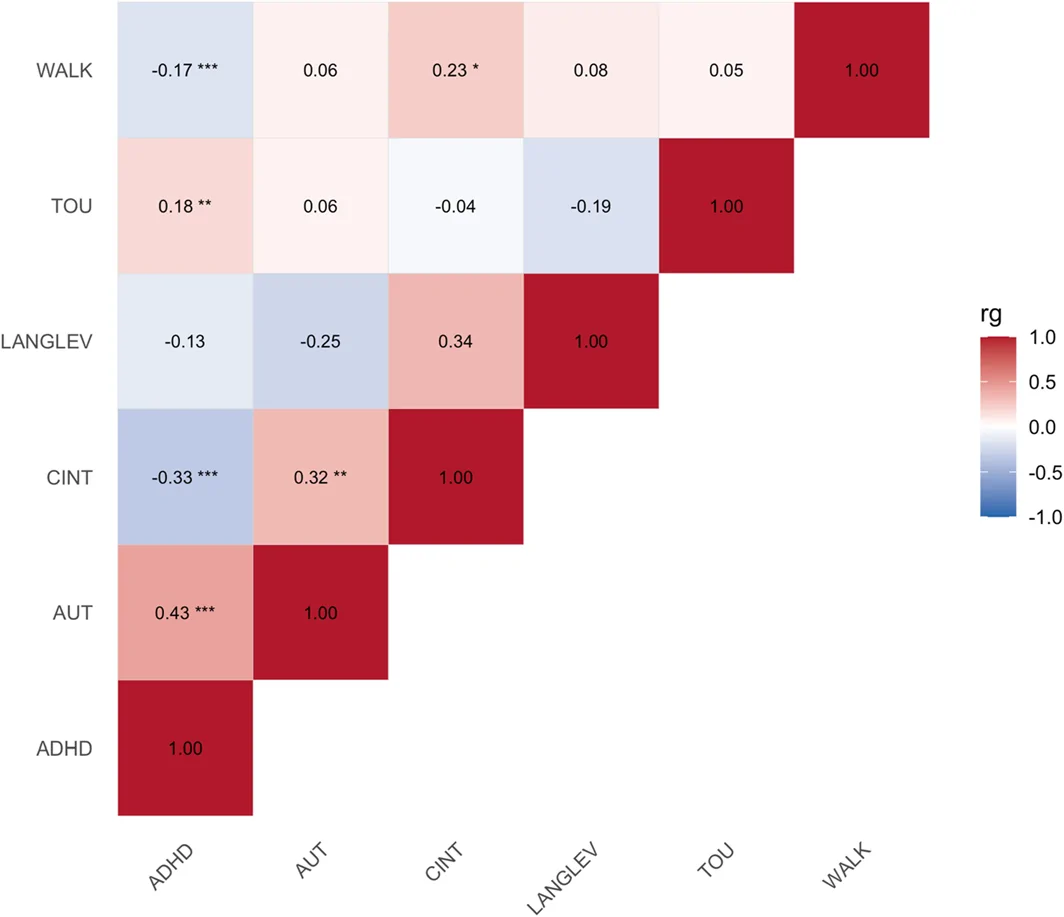
Genetic correlations between neurodevelopmental conditions or their proxies. ADHD, attention deficit/hyperactivity disorder; AUT, autism; CINT, childhood intellectual ability (proxy for intellectual disability); FDR, false discovery rate; LANGLEV, Early expressive vocabulary, a proxy trait for language disorder; TOU, Tourette'’s syndrome; WALK, age at onset of walking, a proxy trait for developmental co‐ordination disorder. Colour indicates the magnitude and direction of the correlation (see colour scale, red for positive and blue for negative). FDR corrected *p* values are indicated: ***p < .001, **p < .01, *p < .05. *r*_g_ refers to genetic correlation.

### Regression models

The regression models carried out using GenomicSEM (Tables 3, 4, 5; Supporting Information S1: Tables S4–S6) show the genetic associations between the conditions after accounting for the effect of the other conditions. Table 3 shows that when ADHD was regressed on the remaining phenotypes, Autism genetic liability significantly positively associated with ADHD (*β* = .51, *p* < .0001, FDR‐corrected), while genetic propensity for DCD (through the proxy of later walking) (*β* = −.19, *p* = .035, FDR‐corrected) and lower intellectual disability (through the proxy of childhood intelligence) (*β* = −.54, *p* = .0004, FDR‐corrected) significantly negatively associated with ADHD liability. Table 4 shows that in relation to Autism genetics, ADHD associated positively and significantly with Autism (*β* = .71, *p* < .0001, FDR‐corrected), DCD (through the proxy of later walking) associated positively and significantly with Autism (*β* = .18, *p* = .047, FDR‐corrected), and lower intellectual disability (through the proxy of childhood intelligence) associated positively and significantly (*β* = .49, *p* = .013, FDR‐corrected). As set out in Table 5, the proxy of childhood intelligence, analysed as an outcome, was negatively associated with ADHD genetics (*β* = −.73, *p* < .0001, FDR corrected) and positively associated with Autism genetics (*β* = .47, *p* < .001, FDR corrected). Results for childhood intelligence should be interpreted with directionality in mind, positive associations with this phenotype indicate overlap with higher intellectual ability, whereas negative associations indicate overlap with greater liability to intellectual disability. No other cross‐trait betas survived FDR correction.

**TABLE 3:** Regression model results for ADHD predicted by other NDCs.

Outcome	Predictor	Standardised beta (conditional genetic correlation)	*p*	FDR corrected *p*‐value (* significant)
ADHD	Autism	.5072	0	.0000*
ADHD	Tourette's syndrome	.2187	.039	.0819
ADHD	DCD (proxy age at onset of walking)	−.1904	.0148	.0345*
ADHD	Language disorder (proxy early expressive vocabulary)	.0984	.4912	.6068
ADHD	Intellectual disability (proxy childhood intelligence)	−.5449	.0001	.0004*

*Note*: Conditional genetic correlations after accounting for genetic influences on covariates in genomic structural equation models. Multiple regression beta estimates obtained from regression in GenomicSEM of ADHD on autism, Tourette's syndrome, DCD, language disorder, and intellectual disability (or proxies where relevant).

Abbreviations: ADHD, attention deficit‐hyperactivity disorder; DCD, developmental co‐ordination disorder; FDR, false discovery rate; NDCs, neurodevelopmental conditions.

*Significant at *p* < .05.

**TABLE 4:** Regression model results for autism predicted by other NDCs.

Outcome	Predictor	Standardised beta (conditional genetic correlation)	*p*	FDR corrected *p*‐value (* significant)
Autism	ADHD	.7079	0	.0000*
Autism	Tourette's syndrome	−.0993	.4185	.5859
Autism	DCD (proxy age at onset of walking)	.1843	.0267	.0467*
Autism	Language disorder (proxy early expressive vocabulary)	−.1602	.2545	.4111
Autism	Intellectual disability (proxy childhood intelligence)	.4857	.0069	.0132*

*Note*: Conditional genetic correlations after accounting for genetic influences on covariates in genomic structural equation models. Multiple regression beta estimates obtained from regression in GenomicSEM of autism on ADHD, Tourette's syndrome, DCD, language disorder, and intellectual disability (or their proxies where relevant).

Abbreviations: ADHD, attention deficit‐hyperactivity disorder; DCD, developmental co‐ordination disorder; FDR, false discovery rate; NDCs, neurodevelopmental conditions.

*Significant at *p* < .05.

**TABLE 5:** Regression model results for intellectual disability (proxy childhood intelligence) predicted by other NDCs.

Outcome	Predictor	Standardised beta (conditional genetic correlation)	*p*	FDR corrected *p*‐value (* significant)
Intellectual disability (proxy childhood intelligence)	ADHD	−.7329	0	.0000*
Intellectual disability (proxy childhood intelligence)	Autism	.4681	.0004	.0009*
Intellectual disability (proxy childhood intelligence)	Tourette's syndrome	.1393	.3691	.5167
Intellectual disability (proxy childhood intelligence)	DCD (proxy age at onset of walking)	−.0998	.3977	.522
Intellectual disability (proxy childhood intelligence)	Language disorder (proxy early expressive vocabulary)	.0889	.6676	.7379

*Note*: Conditional genetic correlations after accounting for genetic influences on covariates in genomic structural equation models. Multiple regression beta estimates obtained from regression in GenomicSEM of intellectual disability on ADHD, autism, Tourette's syndrome, DCD, and language disorder (or their proxies where relevant).

Abbreviations: ADHD, attention deficit‐hyperactivity disorder; DCD, developmental co‐ordination disorder; FDR, false discovery rate; NDCs, neurodevelopmental conditions.

*Significant at *p* < .05.

### Factor analysis

The results of the EFA using odd chromosome data are shown in Supporting Information S1: Tables S7–S9. Details of the different factor models specified from these analyses using even chromosome data are set out in the Supporting Information file (Supporting Information S1: Tables S10–S13). The fit indices for these models are presented in Table 6.

**TABLE 6:** Model fit indices for the confirmatory factor analysis models.

Models (even chromosome data)	AIC	CFI	SRMR
Single factor model	82.4	0.727	0.135
Two factor model	41.1	0.977	0.084
Bi‐factor model	NA	NA	NA
Cholesky model (two uncorrelated factors)	40.3	0.987	0.082

Abbreviations: AIC, akaike information criterion; CFI, comparative fit index; SRMR, standardised root mean square residual.

Confirmatory factor analyses on even chromosome data demonstrated that a single‐factor model fitted poorly according to both absolute and comparative measures (AIC = 82.4, CFI = 0.727; SRMR = 0.135), whereas a two‐factor correlated model had good fit according to absolute measures and a significantly improved fit based on the comparative measure (AIC = 41.1, CFI = 0.977; SRMR = 0.084). A two‐factor Cholesky model showed similarly good fit, with only minimal differences in fit indices compared with the correlated two‐factor model (AIC = 40.3; CFI = 0.987; SRMR = 0.082). Given the very similar fit statistics, the correlated two‐factor and Cholesky models are both presented. This Cholesky model specified that intelligence was modelled as a latent factor loading onto all of the NDCs, with a zero correlation between those NDCs, and a zero correlation between the intelligence latent factor and the neurodevelopmental latent factor (see Figure 3 and Supporting Information S1: Tables S12 and S14). The bifactor model was fully saturated, so conventional fit statistics could not be estimated.

### Genomic factor analysis SEM models

Figures 2 and 3 present the estimates from the two correlated‐factor and Cholesky models, both of which showed similarly good fit in the CFA, using data from both odd and even chromosomes (Supporting Information S1: Tables S14 and S15). Figure 2 displays the results of the two factor model. There is a correlation between the two factors (0.23). DCD, Autism and the proxy of childhood intelligence loading positively onto the first latent factor (0.21, 0.32 and 0.62, respectively), whereas Tourette's syndrome and ADHD loading negatively onto the first latent factor (−0.22 and −0.75, respectively). The second latent factor has positive loadings only with the proxy of expressive vocabulary (0.16), whereas Autism, ADHD and the proxy of childhood intelligence load negatively (−1.02, −0.51 and −0.19, respectively). Figure 3 displays the results of the two factor Cholesky model, where intelligence is a latent factor loading onto all NDCs. There is no correlation between the NDCs and the proxy of childhood intelligence, nor between the two latent factors. The model shows an intelligence related latent factor, which operates as an intellectual disability factor, with the proxy of childhood intelligence loading negatively onto the factor (−0.61) with Autism and the DCD proxy also loading negatively onto the factor (−0.39 and −0.24 respectively). Both ADHD and Tourette's Syndrome loading positively onto this intellectual disability factor (0.67 and 0.13 respectively). The second latent factor, labelled the neurodevelopmental factor, has positive loadings with Autism, ADHD and Tourette's Syndrome (0.74, 0.92 and 0.16 respectively) and the DCD and Language Disorder proxies loading negatively (−0.02 and −0.16 respectively).

**FIGURE 2:**
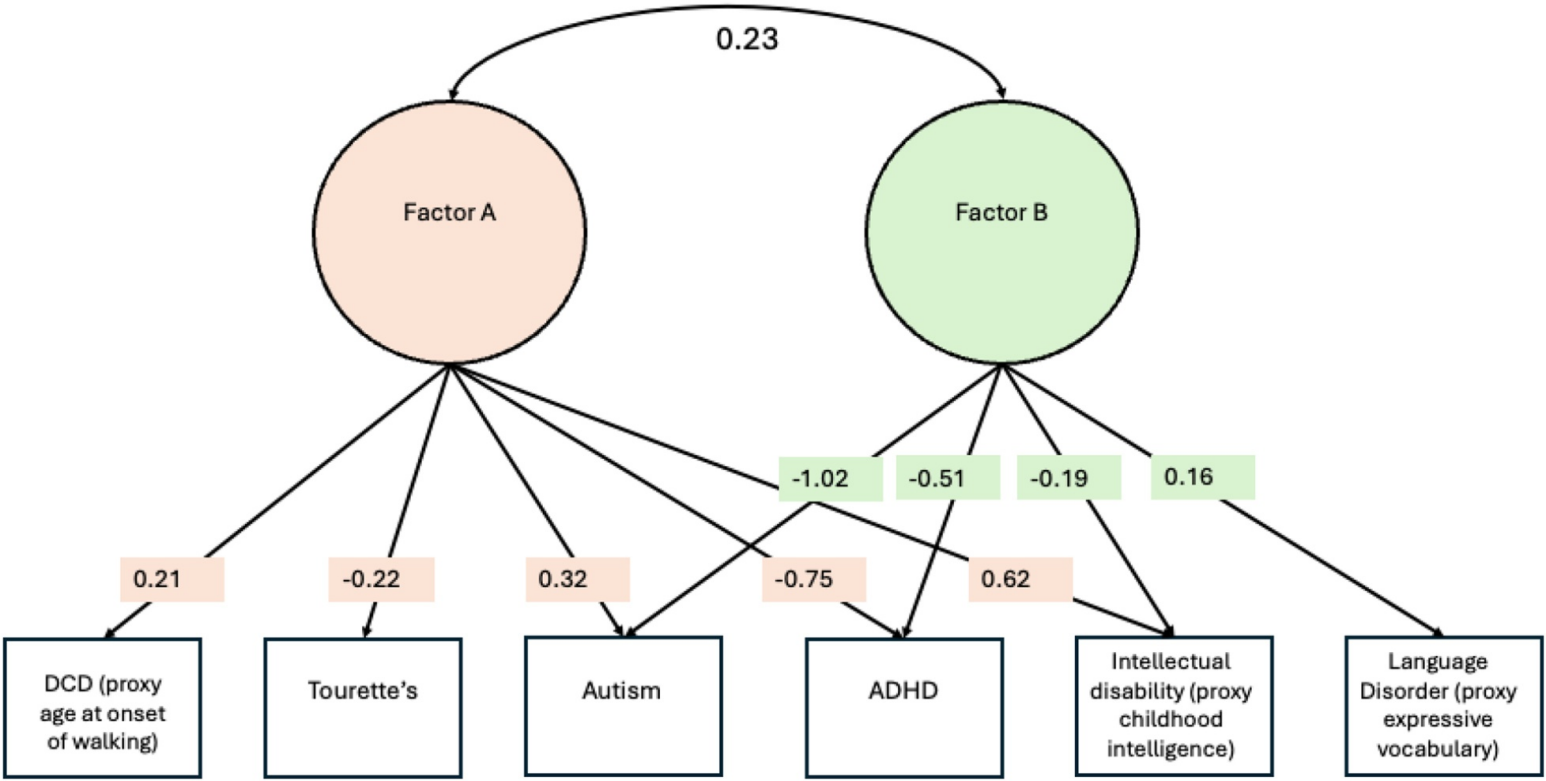
Structural equation model path diagram showing path estimates for two factor (correlated factors) model (using all chromosome data) using genome‐wide association summary statistics for neurodevelopmental conditions (or proxies). Standardised results shown. Regression relationships between the latent factors and the neurodevelopmental conditions are displayed as one‐headed arrows. Covariance relationships between the latent factor or neurodevelopmental conditions are represented as two‐headed arrows. ADHD, attention deficit‐hyperactivity disorder; DCD, developmental co‐ordination disorder.

**FIGURE 3:**
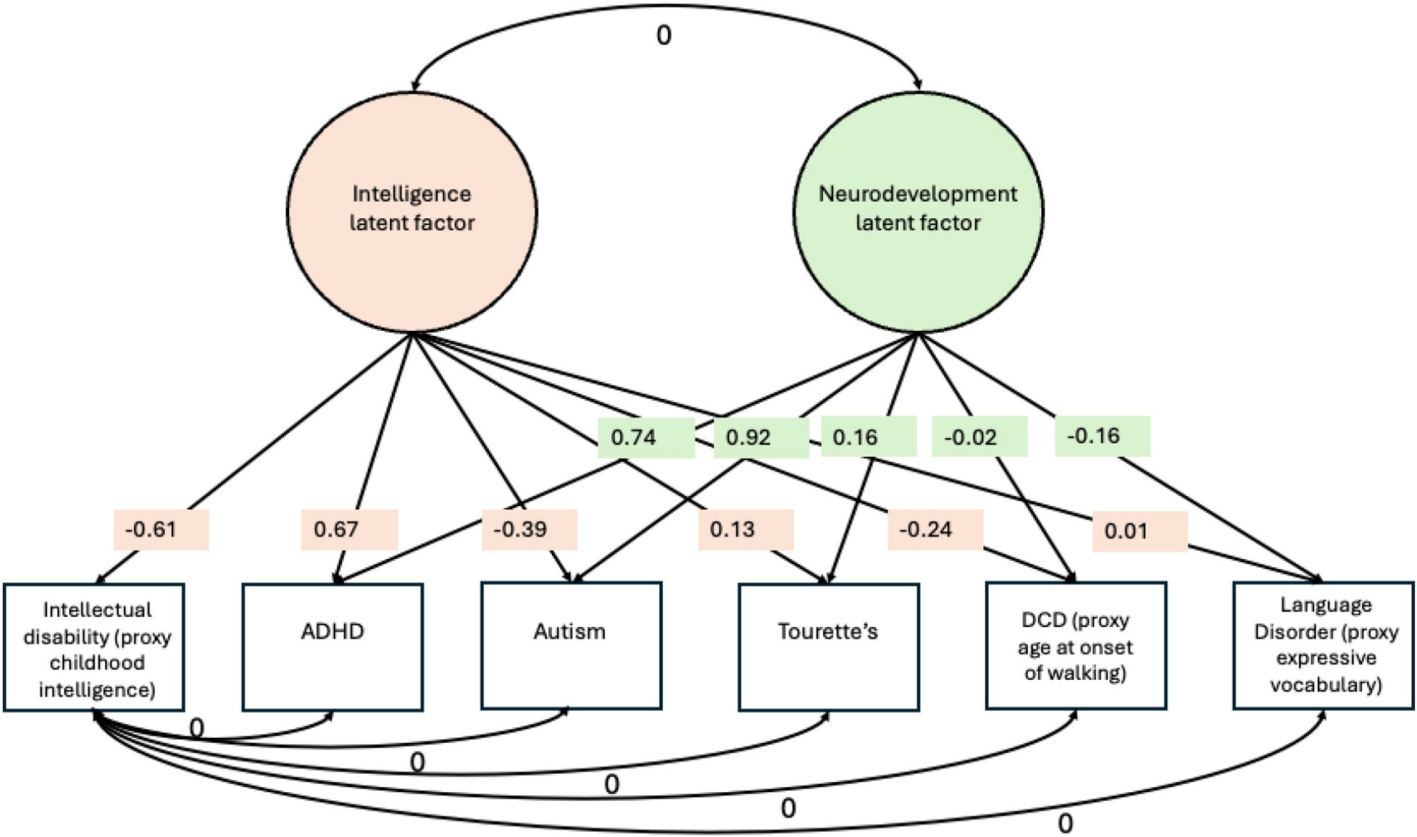
Structural equation model path diagram showing path estimates for two factor Cholesky model (using all chromosome data) using genome‐wide association summary statistics for neurodevelopmental conditions (or proxies). Standardised results from the Cholesky 2 factor model. Regression relationships between the latent factors and the neurodevelopmental conditions are displayed as one‐headed arrows pointing from. Covariance relationships between the latent factor or neurodevelopmental conditions are represented as two‐headed arrows.

## DISCUSSION

This study tested whether a single overarching genetic neurodevelopmental factor (‘*n*’ factor) captures shared liability across neurodevelopmental conditions. Our results do not support the hypothesis that a single factor underlies the shared genetic architecture of autism, ADHD, Tourette's syndrome, intellectual disability, DCD and language disorders, as measured here. A one factor model fitted poorly. Both the correlated two‐factor and the two‐factor Cholesky models fitted the data substantially better than the one‐factor model, but their fit statistics were very similar. Accordingly, these models are best interpreted as plausible alternative representations of a more differentiated genetic structure, rather than as providing clear evidence that one model is superior to the other. In addition, we observed a directionally opposite association of DCD genetics (through its proxy, age at onset of walking) with ADHD versus Autism where the genetics of earlier walking aligned with higher ADHD likelihood whereas later walking aligned with higher Autism likelihood independently of the effect of any other conditions. Although a unitary ‘*n*’ factor was not supported, our findings demonstrate substantial—but selective—genetic overlap across conditions. This is consistent with previous work showing moderate cross‐disorder genetic correlations among neurodevelopmental conditions (Grotzinger et al., 2022; Lee et al., 2019). However, overlap was not universal and certain traits showed weaker or even divergent associations, illustrating that neurodevelopmental genetics cluster into partially overlapping domains rather than one uniform spectrum. This finding is consistent with the common presentation of children with multiple conditions but distinct clinical profiles. Importantly, the multivariate structure was more strongly shaped by ADHD, Autism, and childhood intelligence than by Tourette's syndrome, early expressive vocabulary, or age at onset of walking, indicating that current differences in GWAS signal strength and indicator informativeness likely influenced which dimensions could be resolved in the presented models.

### Overlapping versus specific genetic influences

The present results suggest that the common‐variant genetic architecture of the included neurodevelopmental phenotypes is better characterised as differentiated and selectively overlapping rather than as a single general liability. The poor fit of the one‐factor model indicated that these phenotypes were not well captured by a single latent dimension. However, because the correlated two‐factor and Cholesky models showed very similar fit, the present analyses did not provide a strong basis for favouring a specific two‐domain interpretation, such as distinct intelligence and neurodevelopmental factors. Instead, the more robust inference is that the shared genetic structure is not unitary and that different phenotypes contribute unevenly to the multivariate pattern observed here. Any interpretation of the Cholesky model should be cautious, because its orthogonal structure was imposed to examine the residual pattern after accounting for childhood intelligence, rather than to imply independence between cognitive and non‐cognitive neurodevelopmental liabilities.

Although a single ‘*n*’ factor was not supported by the evidence, we did observe substantial genetic overlap between some neurodevelopmental conditions. For example, genetic correlation analyses indicate that ADHD and Autism share a moderate portion of their common variant architecture consistent with high co‐occurrence rates in clinical populations. In our results, such overlap likely contributed to a common latent component for these disorders. Likewise, ADHD and Tourette syndrome showed shared genetic influences, which concurs with prior studies finding significant overlap of loci across ADHD, autism, and Tourette syndrome (Yang et al., 2021). This corresponds to the observation that more than half of Tourette's patients have comorbid ADHD, and up to 20% have Autism (Yang et al., 2021). Our results indicate that the genetic architecture of neurodevelopmental conditions appears clustered rather than uniform. For example, shared heritable liabilities form overlapping clusters (e.g., an ADHD–Autism cluster), but other aspects remain more disorder specific, such as language disorder and DCD. The presence of both shared and unique genetic factors explains why neurodevelopmental conditions frequently co‐occur yet still manifest distinctive profiles. It refines the ‘general neurodevelopmental spectrum’ concept by showing that some, but not all, of these conditions are linked by common genetic threads. Consequently, the ‘*n*’ factor might be better conceived as a network of interrelated genetic influences, rather than one monolithic dimension.

### Intellectual disability and the ‘*n*’ factor concept

The poor fit of the one‐factor model suggests that childhood intelligence, used here as a proxy for intellectual disability, did not fit comfortably within a unitary general neurodevelopmental factor in these data. However, this should not be overinterpreted as evidence for a definitively separate intelligence factor. The present study included only one cognition‐related proxy phenotype, and the alternative multivariate models that fit better than the one‐factor model were not clearly distinguishable from one another on empirical grounds. Accordingly, the present findings are best understood as indicating that cognitive ability was not well captured by a single common neurodevelopmental factor, rather than as establishing a specific two‐factor structure.

### Genetic influences on early gross motor development differentiate autism and ADHD

We observed a divergence in the genetic association of ADHD and Autism with early motor development, specifically the gross motor milestone age at onset of walking. Earlier walking was linked genetically to increased ADHD likelihood, whereas later walking was genetically associated with higher Autism likelihood. The association with ADHD replicates previous work (Gui et al., 2025), while the Autism finding appears novel and emerges when ADHD effects were accounted for. As noted in Gui et al. (2025), a possible explanation for the ADHD association is that it reflects genetically influenced differences in early activity levels and attention spans. Infants with higher activity levels may have more opportunities for movement practice and motor‐system training, which could contribute to earlier walking onset. This suggests that motor development may differentiate between neurodevelopmental pathways and highlights the value of including developmental milestones in genetic studies of child development.

### Expressive vocabulary and the language domain

Early expressive vocabulary was included in the present study as a proxy for the language‐disorder domain because adequately powered GWAS of clinical language disorder are not yet widely available, whereas expressive vocabulary provides a measurable developmental index of early language acquisition. In this sense, it captures variation in a core language‐related phenotype relevant to later communication development, even though it is not equivalent to a clinical diagnosis. If expressive vocabulary is accepted as a reasonable proxy for language‐related liability, the present findings suggest that this domain was not strongly integrated into the shared multivariate genetic structure observed here. Specifically, expressive vocabulary showed limited evidence of robust shared common‐variant overlap with the other neurodevelopmental phenotypes and contributed less strongly to the latent structure than ADHD, Autism, and childhood intelligence. This indicates that language‐related genetic liability, as indexed here, may be comparatively distinct or may not be well captured by the broad common factors identified in this analysis. At the same time, this interpretation should remain cautious because expressive vocabulary reflects only one early developmental component of the broader language‐disorder construct.

### Methodological considerations and limitations

The study contained several limitations that require discussion. For some conditions we necessarily used proxy phenotypes (such as childhood intelligence for intellectual disability, expressive vocabulary for language disorders and age at onset of walking for DCD) as suitable GWAS data for those conditions were not available. This means the results should be interpreted cautiously as these conditions are not captured specifically. Some GWAS studies for neurodevelopmental conditions were available but not used (such as the DCD GWAS with a sample size of under 5000) as they were underpowered for the analyses. Others, specifically Stuttering and Dyslexia, were not available despite our attempts to access them. Also, the data for all of the conditions was based on European ancestry cohorts which limits the generalisability of the results. It is important that future work replicates this study once GWAS from diverse ancestries are conducted on these conditions. There is also heterogeneity between the samples that make up the GWAS for the different conditions in terms of age, ascertainment and measurement which could influence the results.

An additional limitation is that GWAS discovery sample sizes and signal strength varied substantially across phenotypes. ADHD, Autism, and age at onset of walking were represented by relatively larger GWAS, other phenotypes were based on smaller total samples, which will affect the precision of estimated genetic correlations and their contribution to the multivariate models. Accordingly, while the present results do not support a single overarching *n*‐factor, they should not be taken as definitive evidence regarding the precise number or nature of separable dimensions among non‐cognitive neurodevelopmental phenotypes until larger GWAS are available for all phenotypes.

### Clinical and nosological implications

Our findings carry important implications for how clinicians and researchers understand neurodevelopmental co‐occurrence and classification. In terms of nosology, the uneven genetic sharing across conditions indicates that current diagnostic boundaries are both meaningful and somewhat arbitrary. For example, the genetic overlap between ADHD and Autism may justify their frequent co‐diagnosis and suggests they are closer on a spectrum than to other conditions. These insights could eventually lead to a more refined classification system or dimensional framework where clinicians assess children across multiple dimensions (such as attention–hyperactivity, social communication, motor coordination/tics, cognitive level, etc.), each with partially distinct genetic origins. This approach aligns with transdiagnostic perspectives and the Research Domain Criteria initiative (Insel et al., 2010), aligning classification with underlying biology. Notably, our results highlight that high co‐occurrence rates (e.g., 40% of autistic children having ADHD, or over 50% of ADHD children experiencing motor coordination issues) are not just phenomenological but reflect shared genetic liabilities driving symptom co‐expression. Conversely, recognising divergent genetic factors cautions against overgeneralisation. An intervention effective for one neurodevelopmental condition may not automatically work for another, particularly if the conditions have different genetic bases (for instance, strategies targeting cognitive impairment might help ADHD cases with low IQ but be less relevant for an autistic child with high IQ).

### Future directions

This study improves our understanding of genetic overlap and divergence in child psychopathology, and it raises new questions. Future research should aim to identify specific genetic variants and pathways that underlie the shared versus unique factors highlighted here. For example, genome‐wide analyses of a presumed general neurodevelopmental factor (constructed from overlapping traits) could uncover loci of broad effect, while comparing those to disorder‐specific GWAS hits will clarify which biological pathways distinguish conditions. The question of whether the genetics of neurodevelopmental conditions change with age could be explored through research examining whether there is an age‐dependent aspect to the genetic architecture of these conditions. Additionally, longitudinal studies could assess how polygenic scores for the shared genetic architecture of neurodevelopmental conditions associate with specific developmental trajectories. Our finding regarding the age of walking suggests that early motor development is a meaningful differentiator and it is important to understand more generally how genetic factors on infant and toddler development influence later conditions (Ronald & Gui, 2024). Furthermore, expanding GenomicSEM analyses to include more precisely phenotyped studies, rather than proxies, and incorporating additional neurodevelopmental conditions could further map the landscape of the ‘*n*’ factor versus specific factors. Using GWAS data that include more diverse ancestries (i.e., non‐European) in their discovery samples might increase the applicability and robustness of the analyses. As sample sizes expand, it may become feasible to test more complex models (such as multiple correlated factors or higher‐order factors), which could better represent the hierarchical nature of psychopathology (e.g., a general ‘*p*’ factor with a nested ‘*n*’ factor for neurodevelopmental disorders). Another promising avenue is gene‐environment interplay: genetic architecture might interact with environmental exposures to influence why some children develop multiple neurodevelopmental conditions while others, with similar genetic liability, do not. In sum, further dissecting the genetic architecture of neurodevelopmental conditions will enhance our theoretical models, improve diagnostic criteria, and ultimately inform more nuanced, effective interventions for children and families.

## CONCLUSION

In summary, this study demonstrates that the genetic architecture of neurodevelopmental conditions is complex, and characterised by both a partially shared liability and important distinctions between the conditions. Our results did not support a single latent ‘*n*’ factor in these genetic data. Instead, a two‐factor structure better describes the data. By illuminating how and why these conditions co‐occur or diverge, this work advances our understanding of neurodevelopmental conditions and suggests avenues for early identification and a more dimensional, developmentally informed study. The findings support clinical approaches that are co‐occurrence and dimensionally aware, such as assessing children across domains and diagnostic categories. As GWAS enlarge and cover more neurodevelopmental conditions and traits, future research can build on this genetic evidence and foster more precise diagnostic frameworks and personalised interventions, ultimately improving outcomes for neurodivergent children and their families.

## AUTHOR CONTRIBUTIONS

**Morgan James Morgan**: Writing—original draft; project administration. **Margherita Malanchini**: Writing—review and editing. **Elise B. Robinson**: Conceptualization. **Angelica Ronald**: Writing—review and editing; supervision.

## CONFLICT OF INTEREST STATEMENT

The authors declare no conflicts of interest.

## ETHICAL CONSIDERATIONS

The Research Integrity & Governance Office (RIGO) of the University of Surrey approved this research project on 1 May 2024, and the reference is FHMS 23–24 142 EGA. This study used secondary, de‐identified genomic summary statistics derived from previously published studies that obtained informed consent from all participants; no additional consent was required.

## Supporting information

Supporting Information S1

## Data Availability

The data that support the findings of this study are available from the following sources: the autism, Tourette's syndrome and ADHD summary statistics from the PGC are available at https://pgc.unc.edu/for‐researchers/download‐results/; the age at onset of walking summary statistics are available on figshare at https://doi.org/10.6084/m9.figshare.28071566; the childhood intelligence summary statistics are available from the GWAS Catalogue at https://www.ebi.ac.uk/gwas/home; the early expressive language summary statistics are available from the Max Planck Institute for Psycholinguistics at https://archive.mpi.nl/mpi/.
