# Supplementary material for: Multivariate genetic analyses test for the presence of a general ‘n’ factor underlying neurodevelopmental conditions
Source: JCPP Adv. 2026 Sep 29:e70168. Online ahead of print. doi: 10.1002/jcv2.70168 (PMC13621882; doi:10.1002/jcv2.70168)
Supplement: Supplementary file 1 [file JCV2-9999-e70168-s001.docx]

**Multivariate genetic analyses test for the presence of a general “n” factor underlying neurodevelopmental conditions**

# **Supporting Information**

| **Table S1.** Effective Sample Sizes of GWAS. | | | | | |
| --- | --- | --- | --- | --- | --- |
| **ADHD** | | | | | |
| Cohort | Cases | | Controls | Sample prevalence | Effective sample size |
| iPSYCH1 | 17019 | | 21880 | 0.44 | 38292 |
| iPSYCH2 | 8876 | | 15268 | 0.37 | 22452 |
| deCODE | 8281 | | 137993 | 0.06 | 31249 |
| Barcelona, Spain | 572 | | 425 | 0.57 | 975 |
| Bergen, Norway | 295 | | 202 | 0.59 | 480 |
| Cardiff, UK | 721 | | 5081 | 0.12 | 2526 |
| CHOP, USA | 262 | | 262 | 0.50 | 524 |
| Germany | 487 | | 1290 | 0.27 | 1414 |
| IMAGE-I | 700 | | 700 | 0.50 | 1400 |
| IMAGE-II | 624 | | 1755 | 0.26 | 1841 |
| PUWMa | 563 | | 563 | 0.50 | 1126 |
| Toronto, Canada | 109 | | 109 | 0.50 | 218 |
| Yale-Penn | 182 | | 1315 | 0.12 | 639 |
| **Total effective sample size** | |  |  |  | **103136** |

| **Tourette’s Syndrome** | | | | |
| --- | --- | --- | --- | --- |
| Cohort | Cases | Controls | Sample prevalence | Effective sample size |
| GWAS1 | 969 | 3923 | 0.20 | 3108 |
| GWAS2 | 2711 | 3762 | 0.42 | 6302 |
| GWAS2 FAM | 548 | 597 | 0.48 | 1143 |
| TIC | 591 | 1206 | 0.33 | 1587 |
| **Total effective sample size** |  |  |  | **12140** |
| **Note**. The total effective sample size was calculated as the sum of the effective sample size for each cohort being EffN<-4*v*(1-v)*(cases+controls) where v is sample prevalence. For the Autism GWAS, the summary statistics contained the effective sample size in relation to each SNP so no further calculation was required. | | | | |

| **Table S2.** Heritability of study neurodevelopmental conditions (or their proxy traits). | | |
| --- | --- | --- |
| **Condition/trait** | **H^2^_SNP_** | **SE** |
| Autism | 0.09 | 0.01 |
| ADHD | 0.14 | 0.01 |
| Age at onset of walking | 0.24 | 0.01 |
| Childhood intelligence | 0.27 | 0.05 |
| Infant expressive vocabulary | 0.24 | 0.02 |
| Tourette’s | 0.21 | 0.02 |
| **Note** - H^2^_SNP_ - SNP heritability. SE – Standard Error. Estimates from relevant genome-wide association studies (Age at onset of walking – Gui et al., 2025; Attention Deficit-Hyperactivity disorder (ADHD) – Demontis et al., 2023; Tourette’s – Yu et al., 2019; Infant expressive vocabulary – Verhoef et al., 2024; Childhood intelligence – Benyamin et al., 2014; Autism – LDSC analysis of unpublished data from PGC). | | |

| **Table S3.** Pairwise genetic correlations between neurodevelopmental conditions (or proxy traits). | | | | | | |
| --- | --- | --- | --- | --- | --- | --- |
| **Trait 1** | **Trait 2** | **r_g_** | **SE** | **z** | **p** | **p_FDR** |
| ADHD | AUT | 0.4323 | 0.0572 | 7.557692 | 4.10E-14 | 6.15E-13 |
| ADHD | TOU | 0.1783 | 0.0577 | 3.090121 | 2.00E-03 | 6.00E-03 |
| ADHD | WALK | -0.1713 | 0.0428 | -4.00234 | 6.27E-05 | 4.39E-04 |
| ADHD | LANGLEV | -0.1302 | 0.134 | -0.97164 | 3.31E-01 | 4.69E-01 |
| ADHD | CINT | -0.331 | 0.0844 | -3.9218 | 8.79E-05 | 4.39E-04 |
| AUT | TOU | 0.0556 | 0.0665 | 0.83609 | 4.03E-01 | 4.69E-01 |
| AUT | WALK | 0.0625 | 0.0424 | 1.474057 | 1.40E-01 | 2.63E-01 |
| AUT | LANGLEV | -0.2493 | 0.1391 | -1.79224 | 7.31E-02 | 1.57E-01 |
| AUT | CINT | 0.323 | 0.0923 | 3.499458 | 4.66E-04 | 1.75E-03 |
| TOU | WALK | 0.054 | 0.065 | 0.830769 | 4.06E-01 | 4.69E-01 |
| TOU | LANGLEV | -0.1873 | 0.209 | -0.89617 | 3.70E-01 | 4.69E-01 |
| TOU | CINT | -0.0449 | 0.1452 | -0.30923 | 7.57E-01 | 7.57E-01 |
| WALK | LANGLEV | 0.0823 | 0.1404 | 0.586182 | 5.58E-01 | 5.98E-01 |
| WALK | CINT | 0.2342 | 0.0939 | 2.494143 | 1.26E-02 | 3.16E-02 |
| LANGLEV | CINT | 0.3357 | 0.3071 | 1.093129 | 2.74E-01 | 4.57E-01 |
| **Note***.* r_g_, genetic correlation; SE, Standard Error; z, Z-score; p, p value; False Discovery Rate p value (Benjamini–Hochberg FDR adjustment); ADHD, Attention Deficit-Hyperactivity Disorder, AUT; Autism; TOU, Tourette’s Syndrome; CINT, Childhood intelligence; WALK, Age at onset of walking; LANGLEV, Early expressive vocabulary. | | | | | | |

| **Table S4.** Regression model results for Tourette's Syndrome predicted by other neurodevelopmental conditions (or proxy traits). | | | | |
| --- | --- | --- | --- | --- |
| **Outcome** | **Predictor** | **Standardised beta (conditional genetic correlation)** | **p** | **FDR corrected p-value (* significant)** |
| Tourette's Syndrome | ADHD | 0.4489 | 0.0492 | 0.1033 |
| Tourette's Syndrome | Autism | -0.1461 | 0.4253 | 0.5582 |
| Tourette's Syndrome | DCD (proxy age at onset of walking) | 0.0742 | 0.4371 | 0.5399 |
| Tourette's Syndrome | Language Disorder (proxy infant expressive vocabulary) | -0.1101 | 0.5076 | 0.561 |
| Tourette's Syndrome | Intellectual Disability (proxy childhood intelligence) | 0.2126 | 0.3945 | 0.5523 |
| **Note***.* Conditional genetic correlations after accounting for genetic influences on covariates in genomic structural equation models. Multiple regression beta estimates obtained from regression in GenomicSEM of Tourette's Syndrome on ADHD, Autism, DCD, Language Disorder and Intellectual Disability (or proxies where relevant). *significant at p<0.05. FDR, false discovery rate. ADHD, Attention Deficit-Hyperactivity Disorder; DCD, Developmental Co-ordination Disorder. | | | | |

| **Table S5.** Regression model results for Language Disorder (proxy infant expressive vocabulary) predicted by other neurodevelopmental conditions (or proxy traits). | | | | |
| --- | --- | --- | --- | --- |
| **Outcome** | **Predictor** | **Standardised beta (conditional genetic correlation)** | **p** | **FDR corrected p-value (* significant)** |
| Language Disorder (proxy infant expressive vocabulary) | ADHD | 0.2152 | 0.4965 | 0.6133 |
| Language Disorder (proxy infant expressive vocabulary) | Autism | -0.2509 | 0.2476 | 0.3714 |
| Language Disorder (proxy infant expressive vocabulary) | Tourette's Syndrome | -0.1172 | 0.5093 | 0.5942 |
| Language Disorder (proxy infant expressive vocabulary) | DCD (proxy age at onset of walking) | 0.1294 | 0.3267 | 0.4574 |
| Language Disorder (proxy infant expressive vocabulary) | Intellectual Disability (proxy childhood intelligence) | 0.1445 | 0.6705 | 0.7411 |
| **Note**. Conditional genetic correlations after accounting for genetic influences on covariates in genomic structural equation models. Multiple regression beta estimates obtained from regression in GenomicSEM of Language Disorder (proxy infant expressive vocabulary) on ADHD, Autism, Tourette's Syndrome, DCD, and Intellectual Disability (or proxies where relevant). *significant at p<0.05. FDR, false discovery rate. ADHD, Attention Deficit-Hyperactivity Disorder; DCD, Developmental Co-ordination Disorder. | | | | |

| **Table S6.** Regression model results for DCD (proxy age at onset of walking) predicted by other neurodevelopmental conditions (or proxy traits). | | | | |
| --- | --- | --- | --- | --- |
| **Outcome** | **Predictor** | **Standardised beta (conditional genetic correlation)** | **p** | **FDR corrected p-value (* significant)** |
| DCD (proxy age at onset of walking) | ADHD | -0.3991 | 0.0285 | 0.0599 |
| DCD (proxy age at onset of walking) | Autism | 0.2767 | 0.0427 | 0.0815 |
| DCD (proxy age at onset of walking) | Tourette's Syndrome | 0.0757 | 0.4471 | 0.5523 |
| DCD (proxy age at onset of walking) | Language Disorder (proxy infant expressive vocabulary) | 0.1241 | 0.338 | 0.4732 |
| DCD (proxy age at onset of walking) | Intellectual Disability (proxy childhood intelligence) | -0.1555 | 0.4066 | 0.5337 |
| **Note**. Conditional genetic correlations after accounting for genetic influences on covariates in genomic structural equation models. Multiple regression beta estimates obtained from regression in GenomicSEM of DCD (proxy age at onset of walking) on ADHD, Autism, Tourette's Syndrome, Language Disorder, and Intellectual Disability (or proxies where relevant). *significant at p<0.05. FDR, false discovery rate. ADHD, Attention Deficit-Hyperactivity Disorder; DCD, Developmental Co-ordination Disorder. | | | | |

| **Table S7.** Exploratory factor analysis results with one factor using odd chromosome data. | | | |
| --- | --- | --- | --- |
|  | **Factor Loading** | **Uniqueness** | **Communality** |
| ADHD | 0.998 | 0.005 | 0.995 |
| Autism | 0.413 | 0.83 | 0.17 |
| Tourette's Syndrome | 0.274 | 0.925 | 0.075 |
| WALK | -0.191 | 0.964 | 0.036 |
| Language | -0.016 | 1 | 0 |
| Childhood intelligence | -0.484 | 0.766 | 0.234 |
|  |  |  |  |
| **Note***.* Promax rotation. WALK, Age at onset of walking (proxy for DCD); Language, Early expressive vocabulary (proxy for Language Disorder); Childhood intelligence, proxy for Intellectual Disability. ADHD, Attention Deficit-Hyperactivity Disorder; DCD, Developmental Co-ordination Disorder. | | | |

| **Table S8.** Exploratory factor analysis results two factors using odd chromosome data. | | | | |
| --- | --- | --- | --- | --- |
|  | **Factor 1 Loading** | **Factor 2 Loading** | **Uniqueness** | **Communality** |
| ADHD | 0.976 | 0.220 | 0.005 | 0.995 |
| Autism | 0.216 | 0.977 | 0.005 | 0.995 |
| Tourette's Syndrome | 0.271 | 0.040 | 0.925 | 0.075 |
| WALK | -0.222 | 0.125 | 0.934 | 0.066 |
| Language | 0.015 | -0.147 | 0.978 | 0.022 |
| Childhood intelligence | -0.557 | 0.279 | 0.609 | 0.391 |
|  |  |  |  |  |
| **Note***.* Promax rotation. WALK, Age at onset of walking (proxy for DCD); Language, Early expressive vocabulary (proxy for Language Disorder); Childhood intelligence, proxy for Intellectual Disability. ADHD Attention Deficit-Hyperactivity Disorder; DCD, Developmental Co-ordination Disorder. | | | | |

| **Table S9.** Exploratory factor analysis results with three factors using odd chromosome data. | | | | | |
| --- | --- | --- | --- | --- | --- |
|  | **Factor 1 Loading** | **Factor 2 Loading** | **Factor 3 Loading** | **Uniqueness** | **Communality** |
| ADHD | 0.923 | 0.113 | -0.111 | 0.05 | 0.95 |
| Autism | 0.128 | 0.952 | 0.049 | 0.05 | 0.95 |
| Tourette's Syndrome | 0.347 | -0.046 | 0.134 | 0.902 | 0.098 |
| WALK | -0.284 | 0.187 | -0.072 | 0.924 | 0.076 |
| Language | 0.075 | -0.179 | 0.077 | 0.971 | 0.029 |
| Childhood intelligence | -0.110 | 0.053 | 0.946 | 0.005 | 0.995 |
|  |  |  |  |  |  |
| **Note***.* Promax rotation. WALK, Age at onset of walking (proxy for DCD); Language, Early expressive vocabulary (proxy for Language Disorder); Childhood intelligence, proxy for Intellectual Disability. | | | | | |

| **Table S10.** Confirmatory factor analysis results with one factor using even chromosome data. | | | |
| --- | --- | --- | --- |
| Factor | Indicator | Standardised estimate | p-value |
| F1 | ADHD | -1.010 | 0.000 |
| F1 | AUT | -0.371 | 0.000 |
| F1 | TOU | -0.169 | 0.009 |
| F1 | WALK | 0.133 | 0.007 |
| F1 | LANGLEV | 0.215 | 0.125 |
| F1 | CINT | 0.230 | 0.025 |
| **Note**. F1, Neurodevelopment latent factor; ADHD, Attention Deficit-Hyperactivity Disorder; AUT, Autism; TOU, Tourette’s Syndrome; CINT, Childhood intelligence; WALK, Age at onset of walking; LANGLEV, Early expressive vocabulary. | | | |
| **Table S11.** Confirmatory factor analysis results with two correlated factors using even chromosome data. | | | |
| Factor | Indicator | Standardised estimate | p-value |
| F1 | ADHD | -0.324 | 0.668 |
| F1 | AUT | 0.811 | 0.725 |
| F1 | TOU | -0.185 | 0.026 |
| F1 | WALK | 0.260 | 0.002 |
| F1 | CINT | 0.856 | 0.005 |
| F2 | ADHD | 0.631 | 0.058 |
| F2 | AUT | 1.207 | 0.555 |
| F2 | LANGLEV | -0.256 | 0.068 |
| F2 | CINT | 0.295 | 0.176 |
| F1 | F2 | -0.569 | 0.566 |
| **Note**. F1, First neurodevelopment latent factor; F2, Second neurodevelopment latent factor; ADHD, Attention Deficit-Hyperactivity Disorder; AUT, Autism; TOU, Tourette’s Syndrome; CINT, Childhood intelligence; WALK, Age at onset of walking; LANGLEV, Early expressive vocabulary. F1 as factor and F2 as Indicator shows covariance between latent factors. | | | |

| **Table S12.** Confirmatory factor analysis results with Cholesky model with two factors using even chromosome data. | | | |
| --- | --- | --- | --- |
| Factor | Indicator | Standardised estimate | p-value |
| INTLF | CINT | -0.805 | 0.000 |
| INTLF | ADHD | 0.420 | 0.004 |
| INTLF | AUT | -0.402 | 0.006 |
| INTLF | TOU | 0.069 | 0.608 |
| INTLF | WALK | -0.277 | 0.000 |
| INTLF | LANGLEV | -0.034 | 0.888 |
| F1 | ADHD | 0.816 | 0.033 |
| F1 | AUT | 0.737 | 0.082 |
| F1 | TOU | 0.155 | 0.012 |
| F1 | WALK | -0.063 | 0.244 |
| F1 | LANGLEV | -0.246 | 0.073 |
| **Note**. INTLF, Intelligence latent factor; F1, Neurodevelopment latent factor; ADHD, Attention Deficit-Hyperactivity Disorder; AUT, Autism; TOU, Tourette’s Syndrome; CINT, Childhood intelligence; WALK, Age at onset of walking; LANGLEV, Early expressive vocabulary. | | | |

| **Table S13.** Confirmatory factor analysis results with a correlated bi-factor (two factors) model using even chromosome data. | | | |
| --- | --- | --- | --- |
| Factor | Indicator | Standardised estimate | p-value |
| F1 | ADHD | -0.346 | 0.809 |
| F1 | AUT | 0.455 | 0.691 |
| F1 | TOU | -0.038 | 0.949 |
| F1 | WALK | 0.260 | 0.841 |
| F1 | CINT | 0.854 | 0.760 |
| F2 | ADHD | 0.412 | 0.703 |
| F2 | AUT | 0.149 | 0.592 |
| F2 | LANGLEV | 0.823 | 0.726 |
| F2 | CINT | 0.268 | 0.408 |
| GF | ADHD | -0.843 | 0.154 |
| GF | AUT | -0.622 | 0.965 |
| GF | TOU | -0.171 | 0.869 |
| GF | WALK | 0.090 | 0.912 |
| GF | LANGLEV | 0.568 | 0.868 |
| GF | CINT | 0.171 | 0.838 |
| **Note***.* F1, First neurodevelopment latent factor; F2, Second neurodevelopment latent factor; GF, General latent factor; ADHD, Attention Deficit-Hyperactivity Disorder; AUT, Autism; TOU, Tourette’s Syndrome; CINT, Childhood intelligence; WALK, Age at onset of walking; LANGLEV, Early expressive vocabulary. | | | |

| **Table S14.** Confirmatory factor analysis results with Cholesky two factors using all chromosome data. | | | |
| --- | --- | --- | --- |
| Factor | Indicator | Standardised estimate | p-value |
| INTLF | CINT | -0.608 | 0.000 |
| INTLF | ADHD | 0.674 | 0.000 |
| INTLF | AUT | -0.388 | 0.005 |
| INTLF | TOU | 0.131 | 0.240 |
| INTLF | WALK | -0.236 | 0.000 |
| INTLF | LANGLEV | 0.014 | 0.927 |
| F1 | ADHD | 0.738 | 0.051 |
| F1 | AUT | 0.921 | 0.097 |
| F1 | TOU | 0.158 | 0.008 |
| F1 | WALK | -0.024 | 0.543 |
| F1 | LANGLEV | -0.157 | 0.078 |
| **Note**. INTLF, Intelligence latent factor; F1, Neurodevelopment latent factor; ADHD, Attention Deficit-Hyperactivity Disorder; AUT, Autism; TOU, Tourette’s Syndrome; CINT, Childhood intelligence; WALK, Age at onset of walking; LANGLEV, Early expressive vocabulary. | | | |

| **Table S15.** Confirmatory factor analysis results with two correlated factors using all chromosome data. | | | |
| --- | --- | --- | --- |
| Factor | Indicator | Standardised estimate | p-value |
| F1 | ADHD | -0.754 | 0.246 |
| F1 | AUT | 0.319 | 0.820 |
| F1 | TOU | -0.217 | 0.001 |
| F1 | WALK | 0.211 | 0.000 |
| F1 | CINT | 0.617 | 0.001 |
| F2 | ADHD | -0.509 | 0.180 |
| F2 | AUT | -1.022 | 0.386 |
| F2 | LANGLEV | 0.157 | 0.180 |
| F2 | CINT | -0.187 | 0.219 |
| F1 | F2 | 0.225 | 0.844 |
| **Note**. F1, First neurodevelopment latent factor (Factor A in figure S1); F2, Second neurodevelopment latent factor (Factor B in figure S1); ADHD, Attention Deficit-Hyperactivity Disorder; AUT, Autism; TOU, Tourette’s Syndrome; CINT, Childhood intelligence; WALK, Age at onset of walking; LANGLEV, Early expressive vocabulary. F1 as factor and F2 as Indicator shows covariance between latent factors. | | | |
